# Microbial synthetic community ARC prevents aflatoxin and increases rhizobia‐legume nodulation couplingly

**DOI:** 10.1002/imt2.70125

**Published:** 2026-04-29

**Authors:** Qi Zhang, Tao Wang, Xiaoqian Tang, Xiaofeng Yue, Meijuan Liang, Xiaojun Zhang, Qin Han, Yang Zhou, Peiwu Li

**Affiliations:** ^1^ Key Laboratory of Biology and Genetic Improvement of Oil Crops, Key Laboratory of Detection for Mycotoxins, Ministry of Agriculture and Rural Affairs, Oil Crops Research Institute, Chinese Academy of Agricultural Sciences Wuhan China; ^2^ Hubei Hongshan Laboratory Wuhan China; ^3^ Xianghu Laboratory Hangzhou China

## Abstract

Develop a novel strategy for exploring a dual‐functional microbial synthetic community. Invent the SynCom ARC, which achieves aflatoxin control and rhizobia nodulation induction coupling in peanut. SynCom ARC inhibits *A. flavu*s growth and reduces peanut aflatoxin levels by 85.6% in 4 year field trials. SynCom ARC enhances peanut nodulation and nitrogenase activity, retains active nodules at harvest, and boosts yield without super nodulation penalty in 325 sites of 19 provinces. SynCom ARC inhibits multiple targets in *A. flavus*, recruits and activates nodulation and nitrogen fixation in rhizobia and peanut, and improves photosynthesis and carbon supply for aflatoxin prevention and nodulation induction, balancing yield increase.

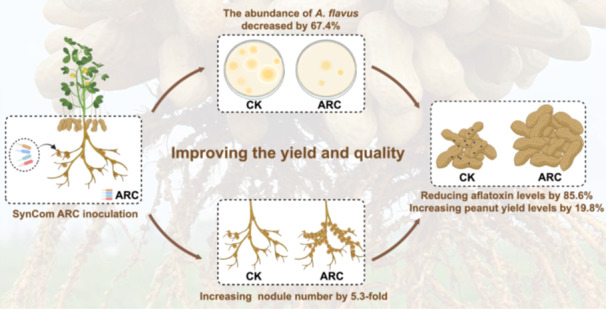

To the Editor,

Legumes contribute approximately one‐third of global dietary protein, playing a vital role in food security and agricultural sustainability [1]. However, Peanut production has long‐term faced two major challenges: aflatoxin contamination and insufficient nitrogen fixation caused by few nodules, which represent key unresolved issues in food and agricultural science, respectively. Since 1960s, aflatoxin has been identified as a highly toxic contaminant of crops such as peanuts and corns, causing significant food losses and health risk worldwide [2]. Although non‐aflatoxigenic strains of *Aspergillus flavus* (*A. flavus*) have been proposed for biocontrol, their application remains limited by safety concerns [3]. Since 1880s, symbiotic nitrogen fixation in legume has been known; increasing nodulation largely relies on rhizobial inoculant. However, super‐nodulation difficultly achieved in legume production [4]. It is generally accepted that nitrogen fixation demands considerable energy from the host; thus, moderate nodulation benefits the plants, whereas excessive nodulation may negatively affect growth [5]. Balancing super‐nodulation and yield, therefore, remains a persistent challenge. The above challenges were independently studied in the past.

To address aflatoxin contamination, continuous monitoring of its distribution and annual variation in Chinese peanuts began in 2009. Systematic analysis of more than 50 agricultural and environmental factors revealed soil‐borne *A. flavus* as the primary source (occupying > 90% of *Aspergillus*) [6]. Since both of the above challenges are focused on soil, we proposed that there could be a technology that can solve them simultaneously. In recent years, synthetic microbial communities have emerged as promising tools for addressing agricultural challenges, offering the potential to simultaneously regulate multiple ecological processes (e.g., pathogen suppression and symbiosis promotion) [7, 8], which inspired us. This study describes the development and validation of ARC (A, aflatoxin prevention and control; R, Rhizobia nodulation induction; C, Coupling), a synthetic non‐rhizobial microbial community that achieves the dual goals of aflatoxin suppression and enhanced nodulation, validated through extensive laboratory and field trials.

## DESIGN AND RATIONALE OF THE SYNTHETIC MICROBIAL COMMUNITY ARC

**Figure 1:**
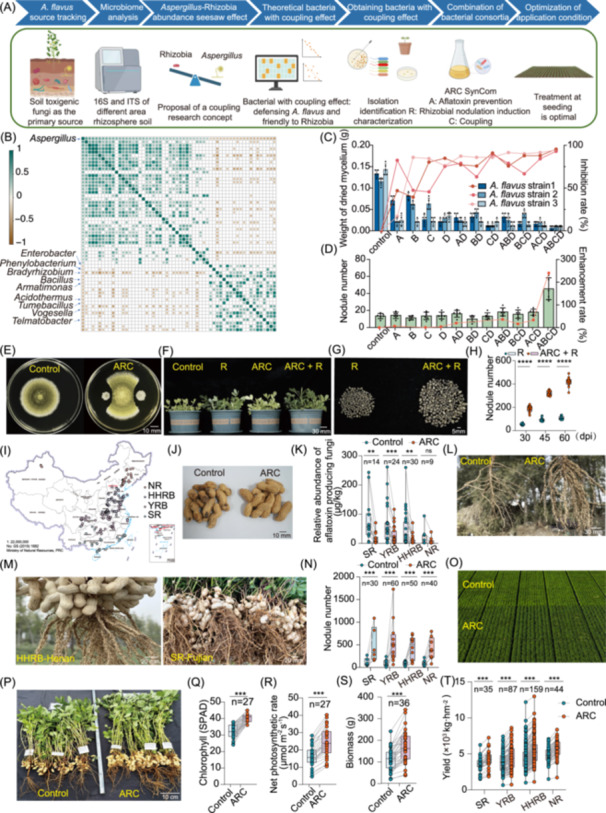
Development and effects of ARC on *A. flavus* suppression, promoting nodulation, nitrogen fixation, and yield increase in peanuts. (A) Workflow for developing ARC (A, aflatoxin prevention and control; R, Rhizobia nodulation induction; C, Coupling). (B) Correlations between rhizosphere microbial genera and *Aspergillus* abundance across regions. (C) Inhibition of *Aspergillus flavus* (*A. flavus*) by different combinations of strains within ARC. A, El17‐4B09; B, Bl13‐2C11; C, Ba13‐20E05; D, Bm14‐5G09. (D) Promoting the nodule number by different strain combinations within ARC. (E) Colony morphology of *A. flavus* under control (left) and ARC treatment (right) in vitro. (F) Peanut plants phenotypes at 60 dpi under different pot treatments (Control; R: Rhizobia alone; ARC: ARC alone; ARC + R). (G) Nodule phenotypes at 60 dpi for R (left) and ARC + R (right) treatments. (H) Nodule number measured at 60 dpi for R and ARC + R (****p < 0.0001, *p* values calculated using Student's *t*‐test). (I) Distribution of national field trial sites. (J) Phenotype: Control (left, showing spotting/rot) versus ARC inoculant‐treated (right, healthy). (K) Abundances of aflatoxigenic fungi in peanuts from four major producing regions. Lines connect paired control/ARC‐treatment points per site, showing aflatoxin reduction by ARC (**p < 0.01, ***p < 0.001, ns, not significant. Statistical analysis used a linear mixed model, and *p*‐values were calculated by Satterthwaite's approximation. SR, Southern Region; HHRB, Huang Huai River Basin; YRB, Yangtze River Basin; NR, Northern Region. (L, M) Close‐up field comparison of roots without versus with ARC inoculant. (N) Nodule number across four major regions (***p < 0.001). Significance was assessed by a generalized linear mixed model with a likelihood ratio test. (O) Aerial view of field plots without versus with ARC. (P) Field comparison of plant canopies. Statistical analysis of chlorophyll II (Q), net photosynthetic rate (R), and biomass (S). Statistical analysis used a linear mixed model, and *p* values calculated by Satterthwaite's approximation. (T) Peanut yield across four regions (***p < 0.001. Statistical analysis used a linear mixed model, and *p* values calculated by Satterthwaite's approximation).

Analysis of the rhizosphere microbiome indicated that the abundance of *Aspergillus* and rhizobia in high‐risk areas and low‐risk areas showed a seesaw phenomenon. This led to the novel proposal of coupling aflatoxin soil source control and promotion of nodulations‐nitrogen fixation (Figure 1A).

A total of 45 soil samples across four major peanut‐producing regions were analyzed using 16S/ITS sequencing (Table S1). To identify bacterial genera with potential coupling effect of defensing *A. flavus* and friendly to rhizobia, a genus‐level abundance matrix was constructed (Table S2). Among the top 200 abundant genera, 37 and 20 showed significantly positive and negative correlations with *Aspergillus* abundance, respectively (Figure 1B and Table S3). Eight of the 20 negatively correlated genera—*Enterobacter*, *Phenylobacterium*, *Bacillus*, *Armatimonas*, *Acidothermus*, *Tumebacillus*, *Vogesella*, and *Telmatobacter*—showed no significant negative correlation with *Bradyrhizobium*, marking them as candidate coupling genera (Figure 1B).

Using culturomics, a total of 127 strains belonging to these eight genera were identified from thousands of isolates in peanut rhizosphere soils or roots collected from different regions (Table S4). Co‐cultured with *A. flavus* in liquid medium, respectively, nine strains demonstrated an aflatoxin inhibition rate exceeding 95%: 7 *Bacillus*, 2 *Enterobacter* (Table S4). Furthermore, co‐culture assays of the 9 strains with *A. flavus* on plates showed that 6 *Bacillus* and 1 *Enterobacter* inhibited fungal growth (Figure S1). However, 3 *Bacillus* inhibited *Bradyrhizobium* (PHNZY‐24‐6) growth (Figure S2). Based on the antifungal activity and rhizobial compatibility, finally, 3 *Bacillus* (Bl13‐2C11, Ba13‐20E05, Bm14‐5G09) and 1 *Enterobacter* (El17‐4B09) were selected for further study.

With the final four strains, several synthetic communities (SynCom) were created and characterized. The result showed that the one with all four‐strains provided optimal against *A. flavus* compared with individual strains and other combinations in laboratory study (Figure 1C). Field experiment showed this combination yielded the highest peanut nodule numbers (Figure 1D). This SynCom was named ARC (A: Aflatoxin prevention and control; R: Rhizobial nodulation induction; C: Coupling). Laboratory tests showed ARC alone did not induce nodulation but promoted significantly more nodules when co‐inoculated with rhizobia (Figure 1E–H).

## ARC CONFIRMS CONSISTENT EFFECTS ON AFLATOXIN SUPPRESSION, NODULATION PROMOTION, AND YIELD ENHANCEMENT IN NATIONWIDE PEANUT TRIALS

After optimization of application conditions such as application period and dosage, a simple protocol was established (about 1.7 × 10^9^ CFU of ARC per hectare, before or during sowing). From 2021 to 2024, ARC was applied in field trials at 325 sites (each site 1–7000 hectares) across China's four major peanut regions (Figure 1I). Paired experiments (with/without ARC) showed ARC significantly reduced peanut surface spotting (Figure 1J) and decreased aflatoxigenic fungal abundance in kernels by 63.2%–71.8% (Figure 1K). After 12 months of natural storage, aflatoxin levels were reduced by an average of 85.6% (81.8%–88.6%) (Figure S3).

Attractively, ARC increased nodule number per plant by an average of 5.3‐fold (range 4.0–8.6‐fold regions) (Figure 1L–N) and nitrogenase activity by 7.4‐fold (Figure S4). Unlike nodules, active ones typically start to decline during pod‐filling; ARC‐treated plants retained abundant active nodules at harvest (Figure S5), continuously supplying nitrogen throughout growth.

ARC applications enhanced peanut photosynthesis and promoted vigorous growth, evident in improved canopy appearance and leaf coloration (Figure 1O,P). Chlorophyll content and photosynthetic rate increased by 27.9% and 54.1%, respectively (Figure 1Q,R). Additionally, root weight, plant biomass, and 100‐pod weight increased by 55.1%, 48.3%, and 6.7%, respectively (Figure 1S and Figure S6). Third‐party verified yield measurements confirmed stable multi‐year and multi‐site field trials increases, with an average enhancement of 19.8% (average 783.5 kg/ha increase of dried pods, range 650.7–856.7 kg/ha) (Figure 1T).

Overall, ARC represents the first successful demonstration in the large‐scale peanut production achieved aflatoxin control simultaneously, super‐nodulation with enhanced nitrogen fixation, and substantial yield increase, balancing the historical contradiction between super‐nodulation and yield. Notably, across nationwide trials with varying soil and climatic conditions, a uniform ARC application method and dosage were used. While minor variations occurred across ecological zones, ARC consistently achieved statistically significant suppression of *A. flavus*, enhanced nodulation and nitrogen fixation, and increased yield—demonstrating its broad adaptability across regions and cultivars.

## ARC RESTRUCTURES THE RHIZOSPHERE MICROBIOME, INHIBITS MULTIPLE TARGETS IN *A. FLAVUS*, RECRUITS AND ACTIVATES NODULATION GENES IN RHIZOBIA

**Figure 2:**
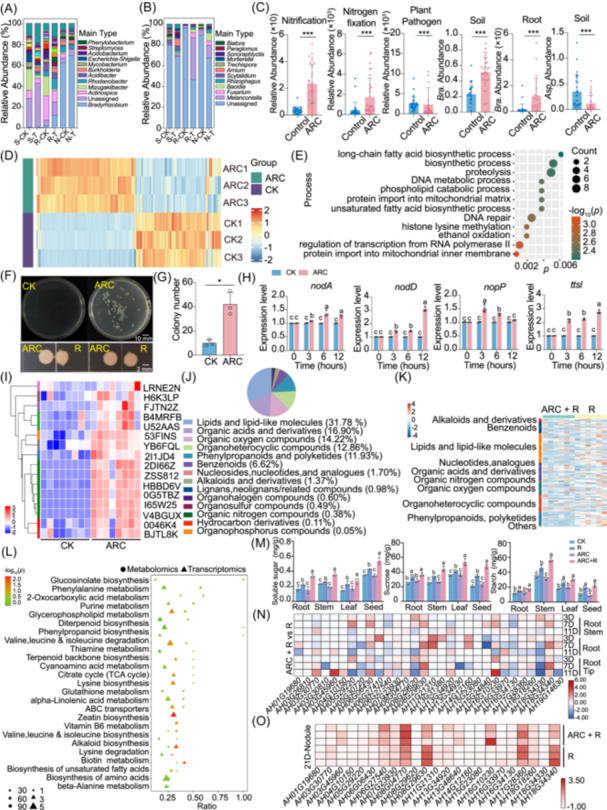
Mechanisms of ARC in defending against *A. flavus* and promoting super‐nodulation and yield balance in peanut. (A) Bacterial and (B) fungal community composition in rhizosphere soil, roots, and nodules after ARC application. (C) Abundances of nitrification, nitrogen‐fixing, and plant‐pathogenic bacteria (ARC/Control) and *Bradyrhizobium* in soil, roots, and nodules. (***p < 0.001, *p* values calculated using non‐parametric Mann–Whitney *U* test). (D) Differentially expressed genes in ARC‐mediated inhibition of *A. flavus*. CK, *A. flavus* alone; ARC, *A. flavus* treated with ARC. (E) GO enrichment analysis of genes involved in ARC‐mediated inhibition of *A. flavus*. (F) Recruitment (top) and confrontation (bottom) assays showing ARC's effect on rhizobia. CK, rhizobia alone; ARC, rhizobia treated with ARC. (G) Quantitative analysis of rhizobial recruitment (*p < 0.05, *p* values calculated using the Wilcoxon rank‐sum test). (H) Expression of rhizobial nodulation‐related genes under ARC treatment (Statistical analysis used two‐way ANOVA). (I) Heatmap of nodulation‐related gene expression in ARC‐treated peanut roots. (J) Summary of metabolites from peanut metabolomics. (K) Heatmap of gene expression in peanut. (L) KEGG enrichment of differentially expressed genes and metabolites. (M) Sugar content across peanut tissues after ARC treatment (Statistical analysis used one‐way ANOVA within each group). Letters denote significant differences (*p* < 0.05). (N) Heatmap of *SWEET* gene expression in roots at early stages. (O) Heatmap of *SWEET* gene expression in nodules at later stages. D, days after inoculation.

To analyze the mechanism of the comprehensive effects, the influences of ARC on the soil microbial structure, *A. flavus*, rhizobia, and peanut were investigated. Sequencing of 163 peanut rhizosphere soil samples showed ARC‐regulated microbial community structure (Figure 2A and Table S5), with 76 bacterial and 20 fungal genera showing variation in abundance. Enrichment analysis indicated nitrogen‐fixing bacteria increase and pathogenic fungi decrease (Figure 2B and Figure S7). Notably, ARC significantly increased *Bradyrhizobium* and decreased *Aspergillus* abundance in rhizosphere soil and roots (*p* < 0.001) (Figure 2C).

Transcriptome sequencing of ARC‐treated *A. flavus* revealed 391 up‐regulated and 273 down‐regulated genes (Figure 2D and Figure S8). GO enrichment analysis showed down‐regulated genes enriched in DNA metabolic/repair, proteolysis, and fatty acid biosynthetic process (Figure 2E and Figure S9), indicating ARC compromises genomic stability, proteostasis, and lipid biosynthesis and metabolism in *A. flavus*.

Co‐culturing of *Bradyrhizobium* with ARC fermentation supernatant yielded three times more colonies than control (Figure 2F,G), indicating ARC promotes rhizobial growth. Gene expression analysis in ARC‐treated *Bradyrhizobium* showed *nodD* expression triple by 12 h, *nodA* transcription increased by 8%–37% within 3–12 h, and T3SS‐related genes *ttsI* and *nopP* were significantly upregulated (Figure 2H). Thus, ARC promotes rhizobial growth and induces nodulation gene expression.

## ARC INDUCES NODULATION AND NITROGEN FIXATION GENES AND CREATES A POWERFUL POSITIVE FEEDBACK LOOP THROUGH IMPROVING PHOTOSYNTHESIS AND CARBON SUPPLY IN PEANUTS

Transcriptomic analyses of ARC inoculant‐treated peanut root showed significant upregulation of nodulation‐related genes (Figure 2I), with 74 up‐ and 39 down‐regulated (Figure S10). Upregulated genes were associated with early infection (e.g., *NIN* and *CYCLOPs*), nodules nutrient/metabolic functions (e.g., *SUS*, *SWEET*, and *PHO84*), and transcription factors (e.g., *DOf* and *SPL*) (Figure 2I), indicating sustained nodule formation and high nitrogen fixation capacity.

ARC inoculation enhanced vegetative biomass, nodulation, and nitrogenase activity (Figure 1N and Figure S4), which was supported by sustained increase in photosynthetic rate, particularly during critical later growth stages (Figure 1O–R and Figure S11). Crucially, transcriptomic and metabolomics analysis revealed ARC reprograms carbon allocation dynamics (Figure 2J–L), specifically upregulation of roots and nodules of genes encoding *SWEET*‐family sugar transporters (Figure 2M,N, and Figure S12), indicating enhanced carbon partitioning to roots (Figure 2O and Figure S13). This creates a powerful positive feedback loop: increased carbon supply supports nitrogen fixation, which in turn promotes growth and photosynthesis.

## FUTURE PERSPECTIVES ON SYNTHETIC MICROBIAL COMMUNITY ARC RESEARCH

Building upon the phenotypic effects and preliminary mechanisms revealed in this study, future research will focus on several key directions: First, there is a need to elucidate how ARC precisely modulates the autoregulation of nodulation (AON) pathways to achieve efficient large‐scale nodulation while avoiding carbon cost penalties. Second, the specific bioactive compounds induced by ARC that suppress pathogens and recruit symbiotic microbes, as well as how it optimizes resource allocation in root architecture and carbon–nitrogen metabolism, remain to be deciphered. Future research on the molecular mechanisms underlying ARC–microbiome–plant interactions should integrate comprehensive analyses of soil physicochemical factors and explore the expansion of ARC application to other crop systems. Finally, exploring the potential of applying ARC‐mediated plant‐microbe interaction mechanisms to non‐leguminous crops will provide novel scientific insights and application strategies for promoting sustainable and high‐yield agricultural development.

In summary, a novel strategy, ARC suppresses *Aspergillus* and triggers super‐nodulation and yield increase, especially balancing historical contradiction between super‐nodulation and yield increase, through inhibiting multiple targets in *A. flavus*, recruits and activates rhizobia nodulation and nitrogen fixation genes, and reprograms plant carbon source dynamics to establish a positive nitrogen‐carbon feedback loop. With successful validation across 325 large‐scale field demonstration sites across China's all four major peanut regions, ARC represents a novel, sustainable, and scalable approach for improving legume production. In the future, we will try to discover the functional molecular modules of ARC–rhizobia–hosts interplay for the expansion of applications in non‐legume crops. This study provides a novel biotechnology for the improvement of food safety, highly efficient legume nitrogen fixation and yield increase, low‐carbon ecology, and sustainable agriculture development, suggesting a potential theoretical evolution from “exclusive rhizobial symbiosis” towards “collaborative nitrogen fixation of non‐symbiotic microorganisms.”

## AUTHOR CONTRIBUTIONS

**Qi Zhang**: Conceptualization; funding acquisition; writing—original draft; resources. **Tao Wang**: Writing—original draft; methodology; formal analysis; supervision. **Xiaoqian Tang**: Conceptualization; writing—review and editing; methodology; supervision. **Xiaofeng Yue**: Writing—original draft; methodology; investigation. **Meijuan Liang**: Supervision; methodology; writing—original draft; investigation. **Xiaojun Zhang**: Writing—review and editing; methodology; software. **Qin Han**: Investigation; writing—original draft; visualization; formal analysis. **Yang Zhou**: Investigation; writing—original draft; visualization; data curation. **Peiwu Li**: Conceptualization; funding acquisition; writing—review and editing; supervision; project administration. All authors have read the final manuscript and approved it for publication.

## CONFLICT OF INTEREST STATEMENT

Qi Zhang and Peiwu Li have filed patents on the SynCom ARC inoculant and its uses. The other authors declare no conflicts of interest.

## ETHICS STATEMENT

No animals or humans were involved in this study.

## Supporting information


**Figure S1.** Plate confrontation assay between 9 candidate strains and *A. flavus*.
**Figure S2.** Co‑culture of 9 candidate strains and *Bradyrhizobium*.
**Figure S3.** The SynCom ARC applications reduced aflatoxin‐producing fungi and thus prevented aflatoxin contamination significantly.
**Figure S4.** The ARC applications in the fields increased the nitrogenase activity by 7.4‐fold in the peanut through the nodulations.
**Figure S5.** Cross‐sectional phenotype of peanut nodules at harvest.
**Figure S6.** Statistical analysis of 100‐pod weight (left) and root weight (right) of peanuts with and without ARC inoculant application in the field.
**Figure S7.** Functional annotation of the peanut rhizosphere microbial community.
**Figure S8.** Volcano plot of differentially expressed genes in *A. flavus* after ARC treatment compared with the control.
**Figure S9.** KEGG enrichment analysis of genes involved in ARC‐mediated inhibition of *A. flavus*.
**Figure S10.** Heatmap depicting the expression of nodulation‐related genes in peanut after SynCom ARC treatment.
**Figure S11.** The comprehensive upregulation of genes encoding PSII, ETC, PSI, and Calvin cycle enzymes enhanced the photosynthetic efficiency observed in ARC‐treated peanuts.
**Figure S12.** Tissue‑specific expression profiles of SWEET genes.
**Figure S13.** qPCR analysis of *SWEET1* and *SWEET2* in roots and nodules in the different stages of root development.

**Table S1.** List of 45 soil sampling sites.
**Table S2.** Genus‐level abundance matrix of 45 soil microbiomes.
**Table S3.** Statistical table of soil microbial taxa with significant correlation to *Aspergillus* abundance at the genus level.
**Table S4.** List of information for 127 strains and their levels of inhibiting aflatoxin.
**Table S5.** List of 163 peanut rhizosphere soil samples collected from the representative demonstration site.
**Table S6.** The following institutions and experts for their help in the field experiments, investigations, and data analysis in the producing regions of peanuts.
**Table S7.** The primers used in this study.
**Table S8.** LC‐MS/MS parameters for the monitoring of AFB1 and CPA.

## Data Availability

The data that support the findings of this study are available in the supplementary material of this article. Sequencing data are deposited in NGDC: PRJCA049496 (https://ngdc.cncb.ac.cn/search/specific?db=bioproject&q=PRJCA049496), PRJCA049495 (https://ngdc.cncb.ac.cn/search/specific?db=bioproject&q=PRJCA049495), CRA039836 (https://ngdc.cncb.ac.cn/gsa/search?searchTerm=CRA039836). The data and scripts used are saved in GitHub https://github.com/xiaojunzhang1/Syncom-ARC/tree/xiaojunzhang1-ARC-data. Supplementary materials (methods, figures, tables, graphical abstract, slides, videos, Chinese translated version, and updated materials) may be found in the online DOI or iMeta Science http://www.imeta.science/.
