# Supplementary material for: Microbial synthetic community ARC prevents aflatoxin and increases rhizobia‐legume nodulation couplingly
Source: Imeta. 2026 Apr 29;5(2):e70125. doi: 10.1002/imt2.70125 (PMC13147931; doi:10.1002/imt2.70125)
Supplement: Supplementary file 1 — Figure S1. Plate confrontation assay between 9 candidate strains and A. flavus. Figure S2. Co‑culture of 9 candidate strains and Bradyrhizobium. Figure S3. The SynCom ARC applications reduced aflatoxin‐producing fungi and thus prevented aflatoxin contamination significantly. Figure S4. The ARC applications in the fields increased the nitrogenase activity by 7.4‐fold in the peanut through the nodulations. Figure S5. Cross‐sectional phenotype of peanut nodules at harvest. Figure S6. Statistical analysis of 100‐pod weight (left) and root weight (right) of peanuts with and without ARC inoculant application in the field. Figure S7. Functional annotation of the peanut rhizosphere microbial community. Figure S8. Volcano plot of differentially expressed genes in A. flavus after ARC treatment compared with the control. Figure S9. KEGG enrichment analysis of genes involved in ARC‐mediated inhibition of A. flavus. Figure S10. Heatmap depicting the expression of nodulation‐related genes in peanut after SynCom ARC treatment. Figure S11. The comprehensive upregulation of genes encoding PSII, ETC, PSI, and Calvin cycle enzymes enhanced the photosynthetic efficiency observed in ARC‐treated peanuts. Figure S12. Tissue‑specific expression profiles of SWEET genes. Figure S13. qPCR analysis of SWEET1 and SWEET2 in roots and nodules in the different stages of root development. [file IMT2-5-e70125-s002.docx]

Supporting information to

**Microbial Synthetic Community ARC Prevents Aflatoxin and Increases Rhizobia-Legume Nodulation Couplingly**

**Running title: ARC inhibits *Aspergillus*, activates rhizobia, and balances super-nodulation and yield**

Qi Zhang ^1, 2^*, Tao Wang^2^, Xiaoqian Tang^1^, Xiaofeng Yue^1^, Meijuan Liang^1^, Xiaojun Zhang^1^, Qin Han^1^, Yang Zhou^1^, Peiwu Li ^1, 3^*

^1^ Key Laboratory of Biology and Genetic Improvement of Oil Crops, Key Laboratory of Detection for Mycotoxins, Ministry of Agriculture and Rural Affairs, Oil Crops Research Institute, Chinese Academy of Agricultural Sciences, Wuhan 430062, China

^2^ Hubei Hongshan Laboratory, Wuhan 430070, China

^3^ Xianghu Laboratory, Hangzhou 311231, China

*Correspondence: zhangqi01@caas.cn (Qi Zhang), peiwuli@oilcrops.cn (Peiwu Li)

**Supplementary Figures**

**Figure S1** **Plate confrontation assay between 9 candidate strains and *A. flavus*.** CK: *A. flavus* cultured alone; each of the 9 candidate strains was inoculated around the *A. flavus* colony.

**Figure S2** **Co‑culture of 9 candidate strains and *Bradyrhizobium.***

Left of the red line: candidate strain placed in direct contact with *Bradyrhizobium*; right of the red line: candidate strain and *Bradyrhizobium* inoculated with spatial separation.


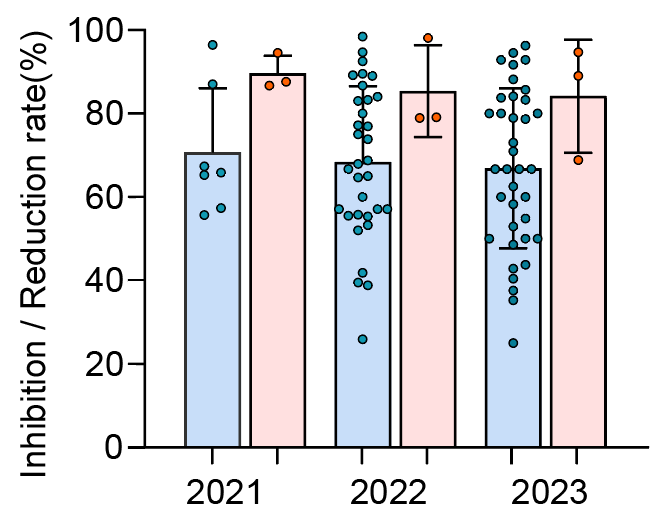


**Figure S3 The SynCom ARC applications reduced aflatoxin-producing fungi and thus prevented aflatoxin contamination significantly.** ARC reduced the relative abundances of aflatoxin producing fungi in peanut fruits and thus prevented aflatoxin of 81.8% – 88.6% (average 85.6%) in peanuts in 2021-2023. Blue: Inhibition rate of *A. flavus*; Orange: Reduction rate of aflatoxin.


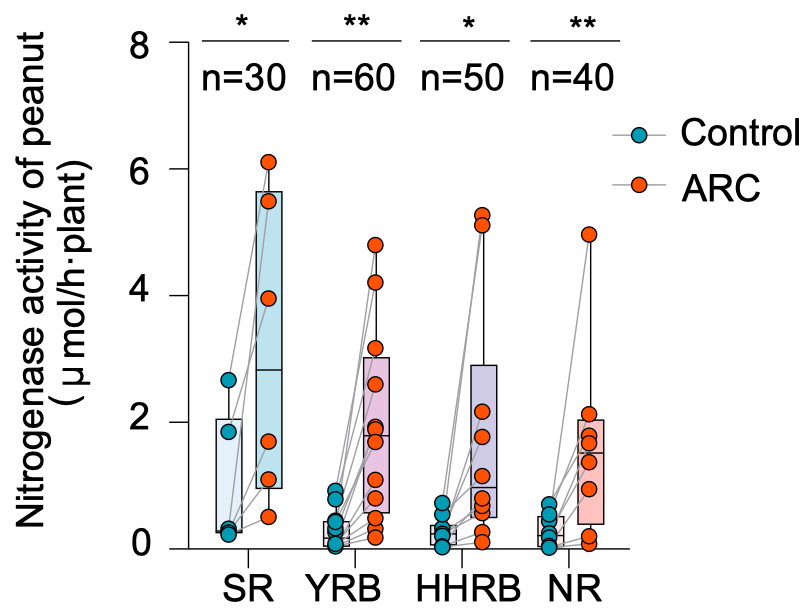


**Figure S4 The ARC applications in the fields increased the nitrogenase activity by 7.4 folds in the peanut through the nodulations** from four major producing regions. Lines connect paired control/ARC-treatment points per site, showing aflatoxin reduction by ARC (*, *p* < 0.05, **, *p* < 0.01, *p* values calculated using linear mixed model and Satterthwaite's approximation). SR, Southern Region; YRB, Huang Huai River Basin; HHRB, Yangtze River Basin; NR, Northern Region.


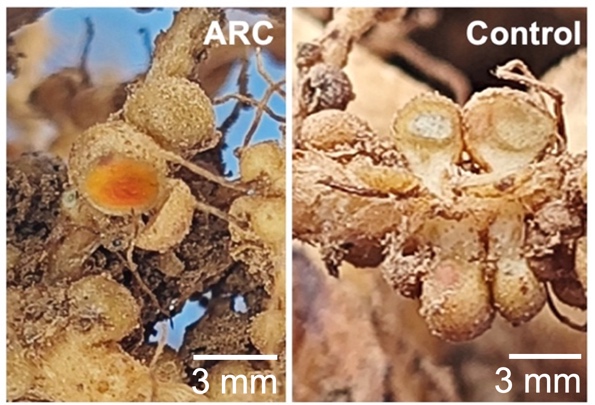


**Figure S5 Cross-sectional phenotype of peanut nodules at harvest.**

Left: ARC treatment, red active nodules with functional leghaemoglobin. Right: Control, yellow-white inactive nodules.


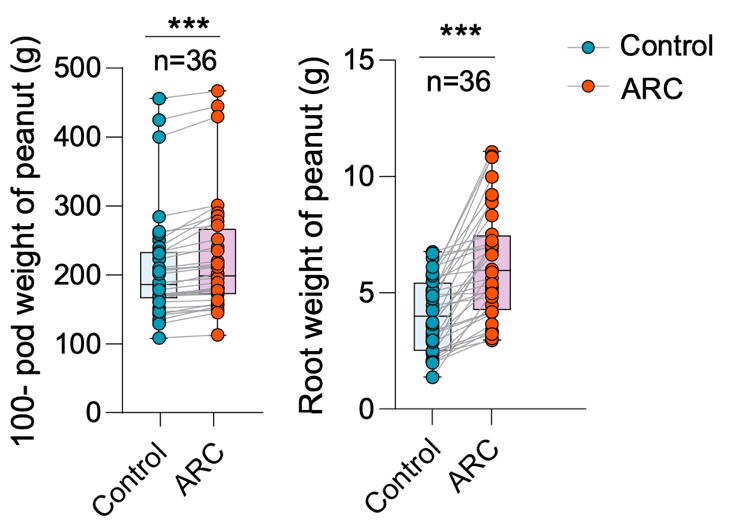


**Figure S6 Statistical analysis of 100-pods weight (left) and root weight (right) of peanuts with and without ARC inoculant application in the field.** Lines connect paired control/ARC-treatment points per site, showing aflatoxin reduction by ARC (*, *p* < 0.05, **, *p* < 0.01, *p* values calculated using linear mixed model and Satterthwaite's approximation). SR, Southern Region; YRB, Huang Huai River Basin; HHRB, Yangtze River Basin; NR, Northern Region.


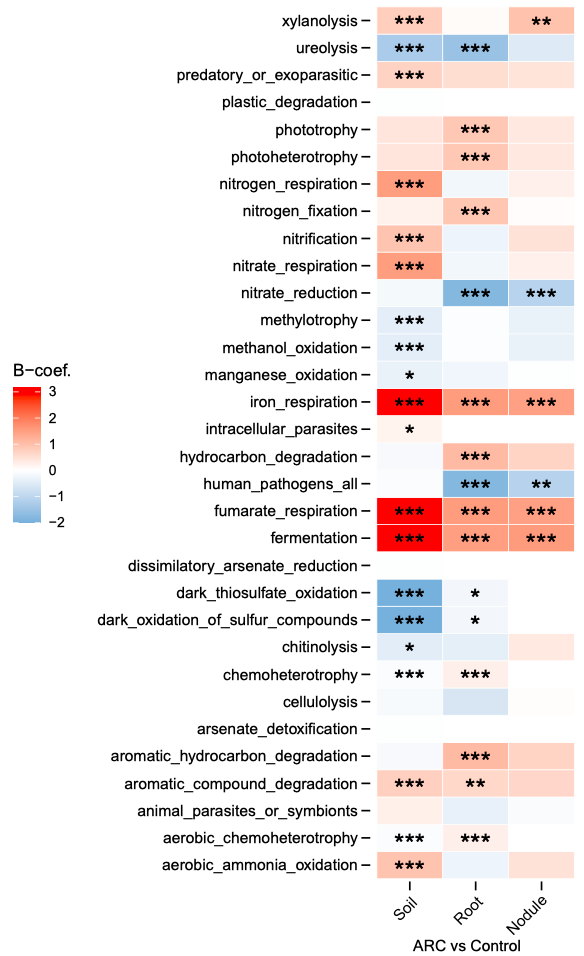

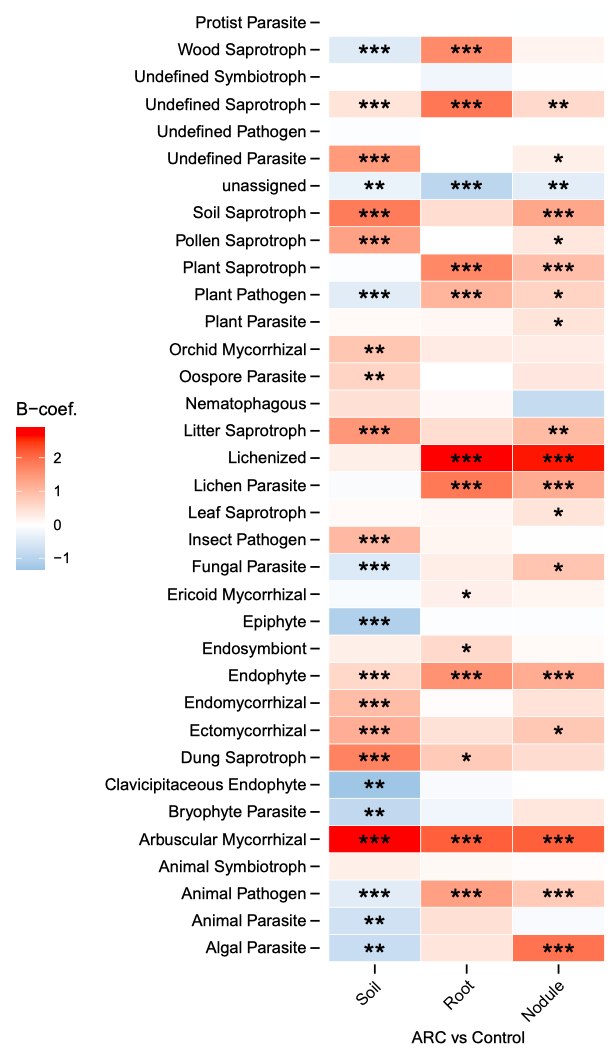


**Figure S7 Functional annotation of the peanut rhizosphere microbial community.** Right: 16S rRNA. Left: ITS. Red indicates upregulation, and blue indicates downregulation. Significance was determined using the FDR with a *p* value < 0.05.


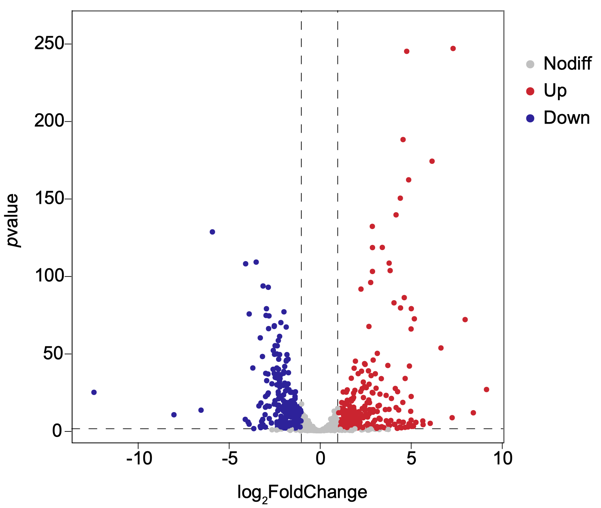


**Figure S8** **Volcano plot of differentially expressed genes in *A. flavus* after ARC treatment compared with the control.** X‑axis: log₂FoldChange; Y‑axis: FDR‑adjusted *p* value. Differentially expressed genes were filtered using an adjusted *p* value < 0.05 and |log₂FoldChange| > 1.


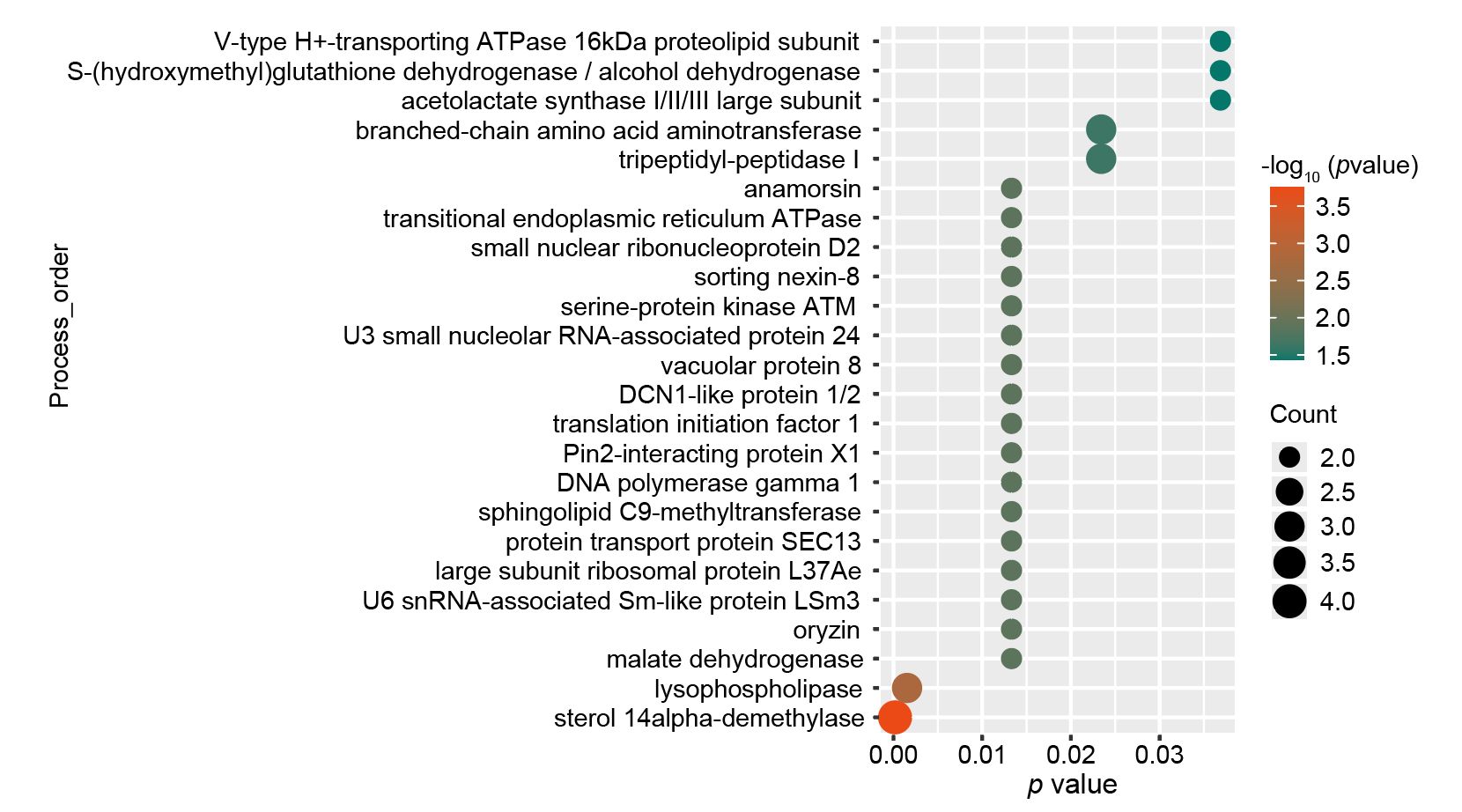


**Figure S9 KEGG enrichment analysis of genes involved in ARC-mediated inhibition of *A. flavus*.** The x-axis represents the *p* value (after FDR adjust), the size of the circles indicates the number of genes, and the color represents the -log_10_ (*p*value).


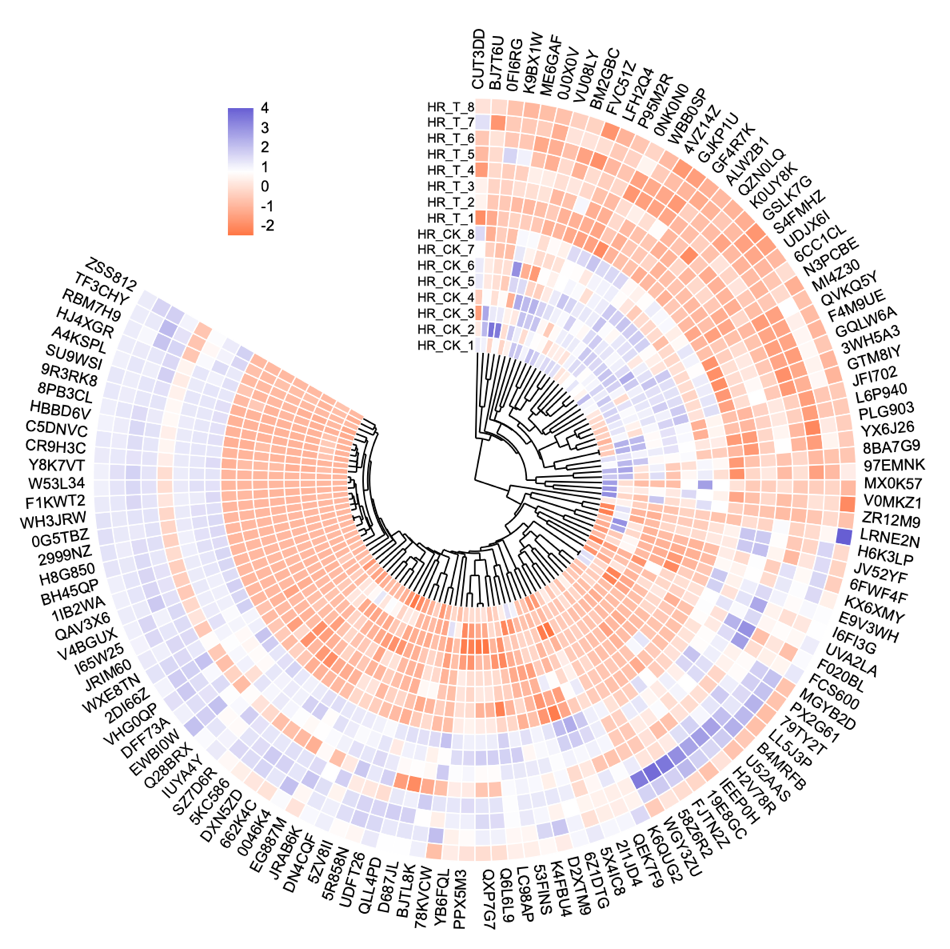


**Figure S10 Heatmap depicting the expression of nodulation-related genes in peanut after SynCom ARC treatment.** The eight outer semicircles represent the ARC treatment, and the eight inner semicircles represent the control. The color represents gene expression abundance.


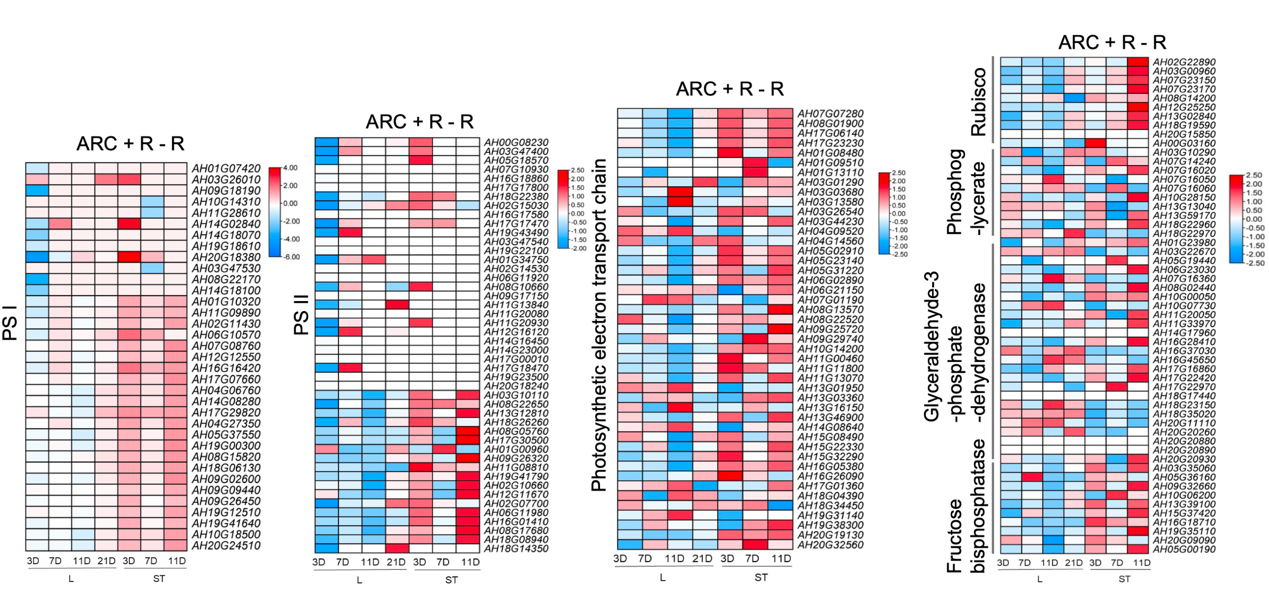


**Figure S11 The comprehensive upregulation of genes encoding PSI, PSII, ETC, and Calvin cycle enzymes enhanced the photosynthetic efficiency observed in ARC-treated peanuts.** The four heatmaps illustrate the differences in gene expression between the ARC + R vs. R, showing the expression changes for genes associated with PSI, PSII, ETC, and the Calvin cycle enzymes from left to right.


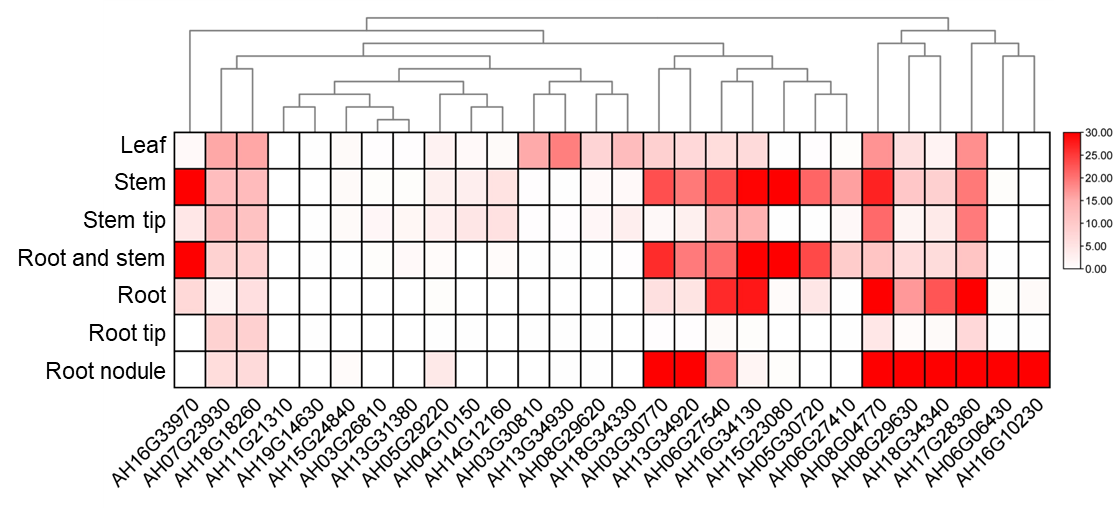


**Figure S12 Tissue‑specific expression profiles of SWEET genes.** Columns represent members of the SWEET gene family in peanut, and rows represent different peanut tissues. The color represents gene expression abundance.

**
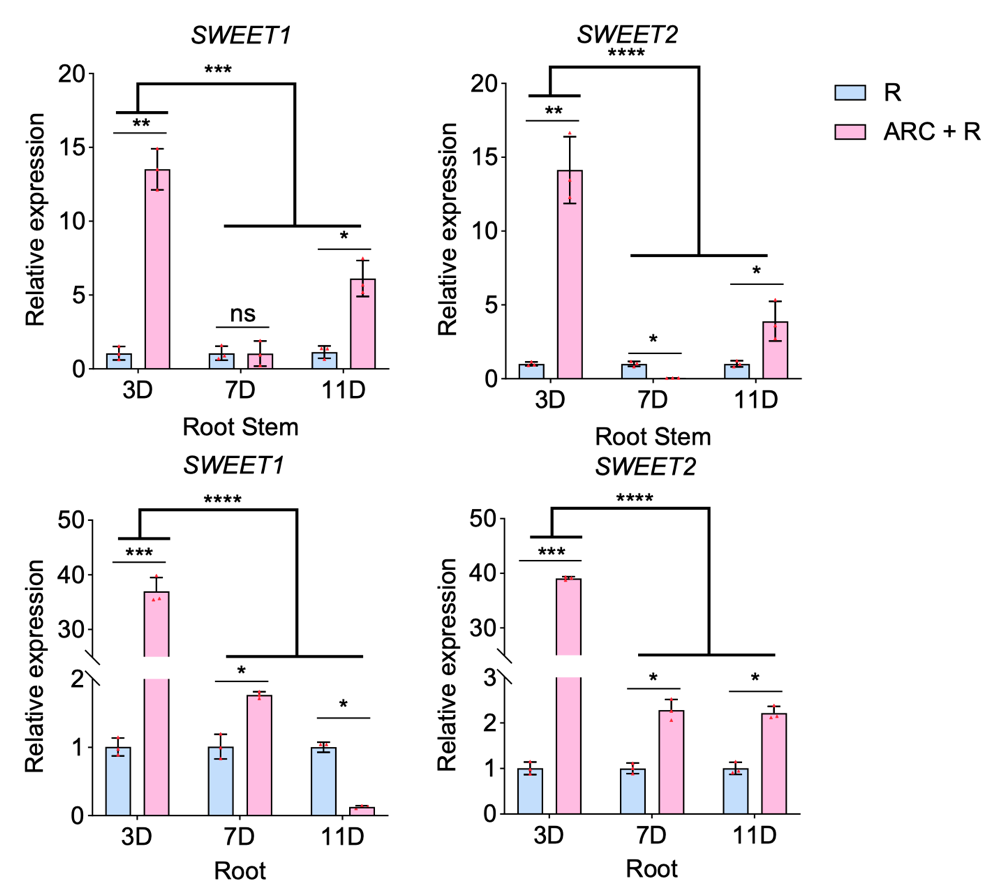
**

**Figure S13 qPCR analysis of *SWEET1* and *SWEET2* in root and root stem in the different stages of root development.** Relative transcript levels of *SWEET1* and *SWEET2* were determined in root and root stem tissues at 3D, 7D, and 11D under the indicated treatments (R and ARC + R). Statistical significance was determined by Student's test or two-way ANOVA followed by Tukey’s multiple comparison test (ns, *p* ≥ 0.05, *, *p* < 0.05, *, *p* < 0.01, ***, *p* < 0.001, ****, *p* < 0.0001).

**Methods**

**1．Collection of rhizosphere soil samples**

Rhizosphere soils (≤ 2 mm from roots) were collected from peanut fields in high-risk areas and low-risk areas across four major peanut-producing regions. Soil samples within 2–3 mm of the root surface was collected using a sterile brush, and 1.0 g was suspended in 9.0 mL of sterile PBS (0.1 M, pH 7.2), vortexed for 2 min, allowed to settle for 5 min, and centrifuged at 8000 ×g for 5 min.

**2. Sequencing and analysis of the rhizosphere microbiome**

Genomic DNA was extracted using a bacterial DNA kit (TIANGEN), and the 16S rRNA gene (V4 region, primers 515F/806R) and ITS (primers ITS1F/ITS2R) amplification, and Illumina MiSeq sequencing were performed. Data were processed with QIIME 2, a genus-level abundance matrix was constructed, correlations among genus abundances were calculated using the Pearson correlation coefficient (r); taxonomic annotation used SILVA (v.138) [1] and UNITE (v.9.0) databases [2]; functional annotation used FAPROTAX and FUNGuild [3]; differential abundance analysis used MaAsLin2 (FDR < 0.05) [4].

**3. Isolation and identification of strains for the biocontrol library**

Samples were collected were was performed as above described. The soil pellet was resuspended in 1 mL PBS, and 20 μL was spread onto LB agar and TSA. Roots and nodules were surface-sterilized sequentially in 75% ethanol (30 s), sterile water (1 rinse), 2% sodium hypochlorite (30 s), and five sterile water rinses, then cut into 3 mm segments or halved before plating on the same media. All plates were incubated at 28°C for 48–72 h. Distinct colonies were purified by repeated four-quadrant streaking. Pure isolates were cultured in LB broth at 28°C with shaking at 180 rpm to the logarithmic phase, mixed 1:1 with 50% sterile glycerol, and stored at −80°C. Bacterial isolates were identified by 16S rRNA gene sequencing (Table S7) [5].

**4. The co-culture method of bacteria strain (biocontrol candidates) and *A. flavus***.

Bacteria strain was grown in LB medium for 24 h at 28 ˚C with shaking at 200 rpm. Cells were counted with a plate counting method. And then *A. flavus* was mixed with the bacteria strain in a 100-mL flask containing 15 mL of peanut medium (potato 200 g/L, dextrose 20 g/L, and peanut flour 1.5 g/L; autoclaved for 20 min at 121 ˚C) [6]. The final concentrations of *A. flavus* was 5×10^5^ spores/mL and that of the bacterial strain was 1×10^7^ colony-forming units (CFU)/mL. Simultaneously, the control treat containing only *A. flavus* in the culture medium was performed. The mixtures were incubated with constant shaking (200 rpm) for 4 d.

**5. Plate confrontation assay of strains with *A. flavus* and *Bradyrhizobium***

The *Bradyrhizobium* strain was grown in TY medium (5 g/L tryptone, 3 g/L yeast extract, 10 mM CaCl_2_, pH 7.0) at 28℃, *A. flavus* was cultured on PDA medium at 28 °C. Four bacterial strains were grown in LB medium at 37 °C. For co‑culture experiments involving the four bacterial strains and all combinations with *A. flavus*, 2 µL of an *A. flavus* spore suspension (1 × 10⁶ spores/mL) was spotted at the center of LA or PDA plates, and 2 µL of a bacterial suspension was then inoculated 1 cm away from the spores. For co‑culture experiments of the four bacterial strains with rhizobia, 2 µL of each bacterial suspension and 2 µL of the rhizobial suspension were spotted onto TY plates at positions 1.0 cm apart. All plates were incubated at 28 °C, and the radial growth of fungal colonies was measured daily for 5 days. The assay was performed in triplicate.

**6. Nodulation assay**

Peanut (*Arachis hypogaea* L.) seeds were surface-sterilized with 75% ethanol for 3 min, rinsed three times with sterile water, imbibed for 2 h, and germinated on sterile vermiculite for 7-10 days. Uniform seedlings were inoculated with 10 mL of *Bradyrhizobium* strain CCBAU suspension (OD_600_ = 0.1), prepared from cultures grown in TY medium at 28°C to logarithmic phase, harvested by centrifugation, and resuspended in sterile water. For microbial treatments, 2 mL of 1% (w/v) ARC inoculant was applied to the rhizosphere at the time of rhizobial inoculation. All plants were grown in a controlled-environment chamber (25–28°C, 55%–65% relative humidity, 16-h light/8-h dark photoperiod.

**7. Demonstration application of the ARC (field trials)**

From 2019 to 2020, we conducted experiments in field to optimize the application conditions such as application period, optimization of using way and dosage. As a result, the application protocol established including mainly 1) The optimal application period was before or during sowing; 2) the appropriate dosage is about 10^14^ CFU of ARC per hectare (about 1.7 x 10^9^ CFU/g of the ARC, application dosage of the ARC is 45 kg/ha); 3) the suitable application way is spreading evenly over the fields by drone before rotary tillage and sowing, or using integrated machinery for trenching, fertilization, and sowing.

**8. Field experimental site**

We developed an optimized protocol for ARC application and then the ARC was applied in all the main peanut producing areas including Southern China, the Yangtze River Basin, the Huang-Huai River Basin, and Northern China (longitude range 85.28 °E–125.79 °E, latitude range 21.06 °N–45.19 °N, altitude range 2 m–1,230 m, soil types including sandy soil, sandy loam, yellow soil, red soil, clay, sandy ginger black soil, etc.). For peanut from 2021 to 2024 with a total of 325 demonstration sites with areas ranging from about 1 to 6,666.7 hectares. At the same time, the control fields without use of ARC have also been set up in the same fields isolated by a field ridge over 20 cm height and over 100 cm width or field ditch over 30 cm depth and over 50 cm width. The varieties and production management measures of the control field and ARC treatment field in the same demonstration site were the same. All these demonstration sites utilized the respective local peanut varieties.

**9. Nitrogenase activity testing**

Peanut plant samples (≥ 5 plants per site) were collected from both control and treatment groups for nitrogenase activity measurements, respectively. These plants were transported back to the laboratory at low temperature using ice packs. The root systems of the plants with roots wrapped in soil were first carefully washed to remove soil and excised. The roots were placed in a 60 mL glass bottle, which was then sealed. Then, a 3 mL of acetylene gas was injected into the bottle, and the samples were incubated at 28°C for 2 h. After incubation, 5 mL of the reaction gas was extracted and transferred into a 20 mL headspace sample vial. Finally, the generated ethylene was measured using a headspace gas chromatography equipped with a hydrogen flame ionization detector (FID) (HS10-GC2030, Shimadzu, Japan). Ethylene amount was quantified using a standard ethylene calibration curve. The ethylene molar amount produced was divided by the reaction time to calculate the nitrogenase activity for each individual plant sample.

**10. Relative chlorophyll content (SPAD values) testing**

From the full-pod to mature stage of peanuts, SPAD values for fully expanded leaves were obtained. From each treatment, 10 plants with similar growth were selected, and their SPAD values were measured using a Minolta SPAD-502 chlorophyll meter during 08:00 am to 11:00 am. To determine the canopy SPAD value, record the SPAD values of all fully unfolded leaves, starting from the bottom and moving towards the top. After completing all leaves measurements, calculate the average SPAD value of all leaves, which will serve as the canopy SPAD value. The results obtained from each treatment were averaged to obtain representative measurements.

**11. Yield measurement**

All yield testing work were completed by authoritative expert groups organized by third-party organizations such as the National Agro-Tech Extension Service Center. During the harvest period of peanut, 3–5 points were taken from each of the ARC treated field and the control field without ARC, with each point covering an area of 5 square meters to over 1,000 square meters for peanut. After picking the fruits, they were weighed and converted into the weight of dried fruits with standard moisture. Finally, the yield increase rates were calculated based on the ARC treated field yield and the control field yield.

**12. Plant growth and nodulation testing**

During the flowering period of peanut, representative plant samples (≥ 5 sites per field) were randomly taken from multiple points in the field of the ARC treatments and that of control without ARC. The number of root nodules was then counted, and the length, weight of fresh roots and aboveground parts, nitrogenase activity.

**13. Photosynthetic rate and relative chlorophyll content (SPAD values) testing**

Photosynthetic rates were tested using the method reported [7]. Briefly, Li-6800 portable photosynthesis system (LI-COR Inc., Lincoln, NE, USA) was used for photosynthetic parameter measurement on the second fully expanded leaf of peanuts from the full-pod to mature stage. SPAD values were tested using the method described [8]. Measurements were conducted on 10 representative plants in each treatment.

**14. *A. flavus* and aflatoxin in peanuts testing**

The sandwich ELISA reported by Wei et al [9]. was used to determine *A. flavus* in peanuts. The peanut samples were crushed into uniform powder, weighed about 20 grams, suspended in a centrifuge tube containing 40 mL phosphate buffer (PBS, 0.01 mol/L), and taken the supernatants for testing.

After harvesting, the peanut samples were stored indoors under natural conditions for 12 months, and then aflatoxins in peanut kernels were detected using ultra-performance liquid chromatography-mass spectrometry (UPLC-MS/MS) [10].

**15. Transcriptomic analysis of *A. flavus* treated with ARC**

RNA was extracted using the RNAprep Pure Plant Plus Kit (Polysaccharides and Polyphenolics‑rich) (DP441; TIANGEN). Library preparation and sequencing were carried out with the VAHTS Universal V6 RNA‑seq Library Prep Kit (NRM604‑02; Vazyme) and the NovaSeq 6000 platform (paired‑end 150 bp reads), respectively. Clean sequencing reads were aligned using HISAT2 [11], and gene expression levels were quantified with StringTie. Differential expression analysis was performed with DESeq2, where genes with |log₂FoldChange| > 1 and *p*_adj_ (BH) < 0.05 were defined as differentially expressed genes.

**16. Rhizobial recruitment assay under ARC treatment**

A capillary assay was used to test *Bradyrhizobium* chemotaxis. *Bradyrhizobium* grown in TY and resuspended in sterile water to OD_600_ = 0.1. Sterile capillary tubes (0.5 mm diameter) were filled with the supernatant of an overnight ARC culture, sealed, and inserted into a 1 mL syringe containing 0.3 mL of bacterial suspension. After 3 h of horizontal incubation in a clean bench, the capillary contents were expelled, diluted, plated on TY agar, and colonies were counted following 24 h of incubation at 28 °C. The assay was carried out in three replicates.

**17. Transcriptomic and metabolomic analysis of plant treated with ARC**

Total RNA extraction and transcriptomic sequencing were performed by Novogene Company. RNA-seq libraries were constructed and sequenced on the Illumina HiSeq platform using a 150-bp paired-end strategy. For metabolomic profiling, metabolites were extracted from the frozen samples and analyzed via Liquid Chromatography-Tandem Mass Spectrometry (LC-MS/MS). Untargeted metabolomic sequencing was conducted by Novogene using a professional LC-MS/MS system to capture a comprehensive profile of the peanut metabolic response.

**18. The ELISA procedure**

1) a 96-well microtiter plate (Costar) was coated with PO8-VHH (*A. flavus* biomarker PO8-specific nanobody, 3.0 μg/mL, 200 μL per well) and left overnight at 4 °C, then was washed with PBS containing 0.05% Tween-20 (PBST);

2) bovine serum albumin solution (3% in PBS, 300 μL per well) was used to block non-specific adsorption sites for 2 h at 37 °C, then was washed with PBST;

3) the supernatants for testing were added into each well for 200 μL and incubated for 1 h at 37 °C, then the plated was washed with PBST;

4) PO8-specific polyclonal antibody from rabbit (2.5 μg/mL in PBS, 200 μL per well) was added and incubated for 50 min at 37 °C, then was washed with PBST;

5) goat anti-rabbit IgG-HRP (Boster) was added into each well with 200 μL and incubated at 37 °C for 50 min, then was washed with PBST;

6) 3, 3’, 5, 5’-tetramethylbenzidine (Sigma-Aldrich) substrate was added with 200 μL per well and incubated at 37 °C for 15 min;

7) then, 50 μL of 2 M H_2_SO_4_ was added to terminate the reaction;

8) the absorbance was measured at 450 nm in SpectraMax i3x multimode microplate reader (Molecular Devices), then the relative concentration of *A. flavus* in peanuts was calculated.

**19. The UPLC-MS/MS method**

The peanut samples were crushed, and 20 g of the peanut sample was added to 60 mL of acetonitrile/water/acetic acid (80:18:2, v/v/v), homogenized for 2 min. Then, the mixture was filtered with glass fiber filter paper. The filtrate was further filtered with a 0.22 μm organic phase filter and injected into the UPLC-MS/MS equipment for determination.

Multiple mycotoxins were identified via UPLC-MS/MS equipment (LCMS-8060, Shimadzu: multiple reaction monitoring modes) methods were compared. Chromatographic separation was performed using a Thermo C18 column (2.7 μm, 10 cm) without a guard column at 40 ◦C. Mobile phase A consisted of 0.05 % formic acid in water, and mobile phase B contained 0.05 % formic acid in ACN. A linear gradient elution program was developed and used mobile phases A and B with the following conditions: 0–2 min, 30–70% B; 2–3 min, 70% B; 3–4 min, 70–30% B; 4–5 min, 30% B. The elution flow rate was set at 300 μL/min, the injection volume was 1 μL, and the run time was 5 min. Spectra were obtained using a triple quadrupole coupled with an electrospray interface (ESI). The MS/MS system was operated using ESI sources in positive or negative mode. The conditions were set as follows: capillary voltage, 3.0 kV for ESI + and − 3.0 kV for ESI−; extractor voltage, 3.0 V; source temperature, 150 ^◦^C; desolvation temperature, 350 ^◦^C. Argon was used as the collision gas (collision cell, 0.8 V), and nitrogen was used as both the nebulizing (50 L/h) and desolvation gases (650 L/h). Data were acquired using LabSolutions software (Version 6.102). The LC-MS/MS parameters for monitoring AFB1 and CPA are shown in Table S8.

**20. Plant tissue sampling for omics analysis**

To investigate the temporal dynamics of the peanut response to microbial inoculation, root and nodule samples were collected at 3, 7, 11, and 21 days post-inoculation (dpi) from two treatment groups: *Bradyrhizobium*-inoculated (R) and ARC *Bradyrhizobium*-inoculated (ARC + R) plants. For transcriptomic analysis, fresh tissues from the root-stem, median root, root tip, and nodules were harvested, with three biological replicates per sample. For untargeted metabolomic analysis, fresh root-stem, median root, and nodule tissues were collected with six biological replicates per sample. All harvested tissues were immediately snap-frozen in liquid nitrogen and stored at –80 °C until further processing. The qRT-PCR primers used are listed in Table S7.

**21. Peanut transcriptomic profiling**

Total RNA was extracted from frozen peanut tissues using a commercial RNA isolation kit following the manufacturer’s instructions. RNA quantity and purity were assessed using a NanoDrop spectrophotometer, only samples with high integrity were used for library construction. RNA-seq libraries were prepared by Novogene (Beijing, China) using poly(A) mRNA enrichment, followed by fragmentation, first- and second-strand cDNA synthesis, end repair, A-tailing, adaptor ligation, and PCR amplification. The resulting libraries were sequenced on an Illumina HiSeq platform with a 150-bp paired-end (PE150) strategy. Raw sequencing reads were pre-processed to remove low-quality adapters, and clean reads were mapped to the peanut reference genome (*Arachis hypogaea* gnm. KYV3 & GCA_003713155.1). Gene expression levels were quantified as FPKM. Differentially expressed genes (DEGs) between groups were identified using DESeq2, with multiple-testing correction applied to control the false discovery rate (FDR). Genes with FDR-adjusted *p* < 0.05 and |log2(fold change)| ≥ 1 were considered significantly differentially expressed. Functional annotation and KEGG pathway enrichment analyses were performed based on the identified DEGs, and pathways with corrected *p* < 0.05 were considered significantly enriched.

**22. Peanut metabolomic profiling (LC–MS/MS)**

Untargeted metabolomic profiling was conducted by Novogene (Beijing, China). Briefly, frozen samples were homogenized under liquid nitrogen, and metabolites were extracted using pre-chilled organic solvent followed by vortexing and sonication. After incubation at low temperature to precipitate proteins, extracts were centrifuged and the supernatants were collected for LC–MS/MS analysis. Quality control (QC) samples were prepared by pooling equal aliquots from each extract and injected periodically throughout the run to monitor instrument stability and analytical reproducibility.

Metabolic extracts were separated by ultra-high-performance liquid chromatography (UHPLC) and analyzed on a high-resolution tandem mass spectrometry system operated. Raw MS data were processed for peak detection, deconvolution, alignment, and normalization to generate a feature matrix (retention time–m/z pairs with corresponding peak intensities). Metabolite identification was performed by matching accurate mass, retention time, and MS/MS fragmentation patterns against public and/or in-house spectral databases. Differentially accumulated metabolites (DAMs) were determined using multivariate analysis PLS-DA and univariate statistics. Metabolites meeting the criteria of VIP ≥ 1, |log2(fold change)| ≥ 1, and FDR-adjusted *p* < 0.05 were defined as DAMs. KEGG pathway annotation and enrichment analyses were performed using the DAMs, and pathways with corrected *p* < 0.05 were considered significantly enriched.

**23. Statistical analysis**

For the correlation analysis of soil microbial abundance, Pearson correlation coefficients were calculated to assess linear relationships between variables. Raw *p*-values obtained from the correlation tests were adjusted for multiple comparisons using the BH-FDR procedure, yielding *q*-values. Correlations with *q* < 0.05 were considered statistically significant. Analyses were performed in R 4.0.0 using the stats package.

For field-measured traits including nitrogenase activity, yield, biomass, 100-pod weight, root weight, net photosynthetic rate, and SPAD values, a linear mixed model was fitted with treatment as a fixed effect and a nested random intercept structure (province/county/site) using the lmer function in R 4.0.0. Degrees of freedom and *p*-values were calculated via Satterthwaite's approximation using the lmerTest package. Significance was set at *p* < 0.05. Notably, nitrogenase activity, yield, and toxigenic fungal abundance were analyzed separately for each of the four major peanut-producing regions.

For nodule count data obtained from the field, which were integer-valued and exhibited overdispersion, they were analyzed using a negative binomial generalized linear mixed model (GLMM) fitted with the glmer.nb function from the lme4 package (version 1.1-26) in R 4.0.0. The model included treatment (CK vs. ARC) as a fixed effect and a nested random intercept structure (province/county/site) to account for the hierarchical experimental design. Significance of the treatment effect was assessed by a likelihood ratio test comparing the full model against a null model without treatment (both retaining the same random structure).

For rhizobial colony counts, the Wilcoxon rank-sum test was employed. Microbial relative abundances were compared between groups using the non-parametric Mann–Whitney U test, with *p* < 0.05 considered statistically significant.

Differential abundance analysis of microbial features was performed using MaAsLin2, a widely used multivariate association method [4]. By analyzing microbial relative abundances with MaAsLin2 while adjusting for potential biases, complementary perspectives on group differences were obtained. In this analysis, latitude was included as a fixed effect and site as a random effect. Multiple testing correction was conducted using the BH procedure, and statistical significance was determined based on an FDR threshold of < 0.05.

For transcriptomic data, differential expression analysis was performed using DESeq2. Genes with |log₂FC| ≥ 1 and BH adjusted *p*-value (*p*adj) < 0.05 were considered significantly differentially expressed. KEGG pathway enrichment analysis was subsequently conducted on the identified DEGs, with pathways considered significantly enriched at padj < 0.05.

For metabolomic data, differentially accumulated metabolites (DAMs) were identified using a combination of multivariate and univariate statistics. Partial least squares discriminant analysis (PLS-DA) was performed, and variable importance in projection (VIP) scores were extracted. Univariate analysis was conducted using Student's *t*-test (two-tailed) followed by BH multiple testing correction. Metabolites meeting the criteria of VIP ≥ 1, |log₂FC| ≥ 1, and BH-adjusted *p*-value (*p*adj) < 0.05 were defined as significantly differentially accumulated.

For time-series qPCR data, two-way analysis of variance (two-way ANOVA) was applied to evaluate the effects of treatment and time point, as well as their interaction.

**Reference**

1.Christian Quast, Elmar Pruesse, Pelin Yilmaz, Jan Gerken, Timmy Schweer, Pablo Yarza, Jörg Peplies, Frank Oliver Glöckner. 2013. “The SILVA ribosomal RNA gene database project: improved data processing and web-based tools.” *Nucleic acids research.* 41 (D1): D590-D596. <https://doi.org/10.1093/nar/gks1219>

2.Rolf Henrik Nilsson, Karl-Henrik Larsson, Andy F S Taylor, Johan Bengtsson-Palme, Thomas S Jeppesen, Dmitry Schigel, Peter Kennedy, Kathryn Picard, Frank Oliver Glöckner, Leho Tedersoo, Irja Saar, Urmas Kõljalg, Kessy Abarenkov. 2019. “The UNITE database for molecular identification of fungi: handling dark taxa and parallel taxonomic classifications.” *Nucleic Acids Research*, 47(D1): D259–D264. https://doi.org/10.1093/nar/gky1022

3.Nhu H. Nguyen, Zewei Song, Scott T. Bates, Sara Branco, Leho Tedersoo, Jon Menke, Jonathan S. Schilling, Peter G. Kennedy. 2016. “FUNGuild: an open annotation tool for parsing fungal community datasets by ecological guild.” *Fungal Ecology.* 20:241–248. <https://doi.org/10.1016/j.funeco.2015.06.006>

4.Himel Mallick, Ali Rahnavard, Lauren J McIver, Siyuan Ma, Yancong Zhang, Long H Nguyen, Timothy L Tickle, George Weingart, Boyu Ren, Emma H Schwager, Suvo Chatterjee, Kelsey N Thompson, Jeremy E Wilkinson, Ayshwarya Subramanian, Yiren Lu, Levi Waldron, Joseph N Paulson, Eric A Franzosa, Hector Corrada Bravo, Curtis Huttenhower. “Multivariable association discovery in population-scale meta-omics studies.” *PLoS Computational Biology*. 17(11):e1009442. https://doi.org/10.1371/journal.pcbi.1009442

5.WILLIAMG WEISBURG, SUSANM BARNS, DALEA PELLETIER, DAVIDJ LANE. 1991. “16S ribosomal DNA amplification for phylogenetic study.” *Journal of Bacteriology* 173: 697–703. <https://doi.org/10.1128/jb.173.2.697-703.1991>

6. Yang, Xiaona, Qi Zhang, Zhi Yuan Chen, Hongxia Liu, Peiwu Li. 2017. “Investigation of Pseudomonas fluorescens strain 3JW1 on preventing and reducing aflatoxin contaminations in peanuts.” *Plos One* 12: e0178810. <https://doi.org/10.1371/journal.pone.0178810>

7. Li, Jian, Kengo Yokosho, Hong Liao, Hong Rui Cao, Zhi Chang Chen. 2020. “Diel magnesium fluctuations in chloroplasts contribute to photosynthesis in rice.” *Nature Plants* 6: 1–12. <https://doi.org/10.1038/s41477-020-0686-3>

8. Ling, Qihua, Huang Paul Jarvis. 2011. “Use of a SPAD-502 meter to measure leaf chlorophyll concentration in *Arabidopsis thaliana*.” *Photosynthesis Research* 107: 209–214. <https://doi.org/10.1007/s11120-010-9606-0>

9.Wei, Xiao, Qi Zhang, Wen Zhang, Hui Li, Peiwu Li. 2020. “Improving the sensitivity of ELISA by large-capacity reaction system of aflatoxigenic fungi-biomarker in agro-products. ” *Scientia Agricultura Sinica* 53, 1473–1481. <https://www.chinaagrisci.com/CN/Y2020/V53/I7/1473>

10. Hu, Xiaofeng, Pengfei Zhang, Du Wang, Jun Jiang, Xiaomei Chen, Yong Liu, Zhaowei Zhang, Ben Zhong Tang, Peiwu Li. 2021. “AIEgens enabled ultrasensitive point-of-care test for multiple targets of food safety: aflatoxin B-1 and cyclopiazonic acid as an example.” *Biosensors Bioelectronics* 182: 113188. <https://doi.org/10.1016/j.bios.2021.113188>

11. Kim, Daehwan, Joseph M. Paggi, Chanhee Park, Christopher Bennett, Steven L. Salzberg. 2019. “Graph-based genome alignment and genotyping with HISAT2 and HISAT-genotype.” *Nature Biotechnology* 37: 907–915. <https://doi.org/10.1038/s41587-019-0201-4>
